# Animal Species Recognition with Deep Convolutional Neural Networks from Ecological Camera Trap Images

**DOI:** 10.3390/ani13091526

**Published:** 2023-05-02

**Authors:** Sazida Binta Islam, Damian Valles, Toby J. Hibbitts, Wade A. Ryberg, Danielle K. Walkup, Michael R. J. Forstner

**Affiliations:** 1 Ingram School of Engineering, Texas State University, San Marcos, TX 78666, USA; dvalles@txstate.edu; 2 Natural Resources Institute, Texas A&M University, College Station, TX 77843, USA; 3 Biodiversity Research and Teaching Collections, Texas A&M University, College Station, TX 77843, USA; 4 Department of Biology, Texas State University, San Marcos, TX 78666, USA

**Keywords:** camera trap, endangered species, image classification, deep learning, machine learning, convolutional neural network, image augmentation, snake, lizard, toad

## Abstract

**Simple Summary:**

The disappearance of many reptiles and amphibian species is directly and indirectly connected with habitat modification, introducing invasive species, disease, pollution, and climate change. Monitoring endangered species is essential for conservation action to mitigate the predominant threats to endangered species. According to wildlife researchers, visual information provides definitive evidence of an animal’s distribution patterns and activity within an environmental context. At the Department of Biology and The Ingram School of Engineering at Texas State University and Texas A&M University, we collaborated on a camera trap project to monitor species from images in Texas, USA. We developed a framework to analyze the acquired images using artificial intelligence-driven technology and computer vision methods. The project aims to classify three broad groups of herpetofaunal species (i.e., toads/frogs, lizards, and snakes) from camera trap images with deep learning architectures. The experiment includes balancing the imbalanced dataset, investigating several image preprocessing techniques, and augmentation procedures. The classification results present tremendous prospects for automated species identification from challenging biological image data. The research outcome is essential to the academic community and wildlife researchers for conservation and monitoring purpose.

**Abstract:**

Accurate identification of animal species is necessary to understand biodiversity richness, monitor endangered species, and study the impact of climate change on species distribution within a specific region. Camera traps represent a passive monitoring technique that generates millions of ecological images. The vast numbers of images drive automated ecological analysis as essential, given that manual assessment of large datasets is laborious, time-consuming, and expensive. Deep learning networks have been advanced in the last few years to solve object and species identification tasks in the computer vision domain, providing state-of-the-art results. In our work, we trained and tested machine learning models to classify three animal groups (snakes, lizards, and toads) from camera trap images. We experimented with two pretrained models, VGG16 and ResNet50, and a self-trained convolutional neural network (CNN-1) with varying CNN layers and augmentation parameters. For multiclassification, CNN-1 achieved 72% accuracy, whereas VGG16 reached 87%, and ResNet50 attained 86% accuracy. These results demonstrate that the transfer learning approach outperforms the self-trained model performance. The models showed promising results in identifying species, especially those with challenging body sizes and vegetation.

## 1. Introduction

Remote cameras or camera traps are a revolutionary tool in the field of wildlife ecology and conservation monitoring [1] that has become more important in parallel to advances in the technology itself. This powerful tool captures an overwhelming number of images, each potentially providing a rich set of information about the presence of an animal in a strategic study area [1], its population size, and community interactions [2]. Researchers can gather biographical and critical evidence remotely and without interference from human observation [2,3]. The acquired raw images can be stored for future investigation [3,4]. Furthermore, sample images provide additional detection information, including precise date, time, and ambient conditions at the time of imaging [5].

As a group, reptiles and amphibians, collectively known as herpetofauna [6], need urgent conservation consideration and effective monitoring action [7]. Reptile diversity is declining globally [6], and nearly one-third of amphibian species are now considered threatened worldwide [8]. Assessments of the camera trapping literature reveal that camera traps have been primarily applied in studying mammals, birds [9,10], and fish [10]. Burton et al. [11] studied 266 camera trapping publications from 2008 to 2013 and observed that the experiments examined mammals (94.8%) and birds (11.9%), with fewer studies focused on reptiles (1.1%) and nearly none on amphibians (0.74%). Camera trapping presents obstacles with reptiles and amphibian monitoring consequent of ectothermy (relatively small or negligible internal physiological sources of heat) and small body size [10].

However, the camera trap monitoring for squamates (snakes and lizards as a group) has recently expanded [9] and is often used to capture behavior or habitat use [10]. Although camera trap surveys are increasing in possibilities [1], processing camera trap images requires manual review, resulting in immense time investment [12]. It is challenging to automatically process the recorded images and identify the species from a photograph as samples suffer from the underlying significant intraclass variation among species, unpredictable poses, lighting brightness variation, motion blurriness, and cluttered background [13,14,15], all exacerbated by natural camouflage effects. Furthermore, partially displayed body fragments and issues with significantly distant or very close targets from the camera lens create obstacles in collecting objective classification features [13].

Most, if not all, of these issues, are familiar to computer vision researchers. To address these concerns, an automatic classification structure has been emphasized in research with computer vision and machine learning techniques. Our work provided insights into deep learning architectures in species classification tasks in biological studies. Furthermore, we optimized self-trained deep learning and machine vision assignments for a challenging camera trap image dataset. The aim was to build a framework that would recognize frogs/toads, snakes, and lizards by classifying them within their group from a large given camera trap dataset.

### 1.1. Deep Learning Solutions

Deep learning is a richer structure of a neural network simulating the information processing patterns of the human brain [16,17]. These multilevel structures extract comprehensive information from input data such as patterns, speech, images, and other applications. Computer vision performance has improved due to the remarkable advances in device capabilities such as memory capacity, power consumption, image sensor resolution, and optics [18]. Moreover, data accessibility and powerful graphics processing unit (GPU) card availability has played a vital role in solving large and complex problems [19]. All these facilities fueled the research towards continuously advancing software algorithms, consecutively improving computational architectures over time [18,19].

In image classification, the core task is to assign a label to an image from a predefined set of possible categories [16,20]. Deep learning algorithms analyze input images to discover relevant patterns in the dataset and classify a new datapoint within their class label [16]. Using big data and plentiful computing resources, convolutional neural networks (CNNs), a deep learning method, make progress on prediction performance with super-human accuracy [19].

Deep convolutional neural networks (DCNN) architectures have continuously evolved in object classification, object localization, and object detection with thousands of categories from the ImageNet dataset through the ImageNet Large Scale Visual Recognition Challenge (ILSVRC) annual competition [12,21]. AlexNet, ZF Net, GoogLeNet, VGGNet, and ResNets architectures were developed by the winner or runner-up teams of the ImageNet competition from ILSVRC2012 to ILSVRC2015, consecutively showcasing the progress in terms of state-of-the-art performance [12,21]. In recent years, considerable research has been performed using pretrained architectures (transfer learning techniques) with camera trap datasets, as described below.

### 1.2. Related Work

A survey of the recent literature suggests that substantial research on automated species identification applying machine learning techniques has been conducted, although primarily for mammals and birds [11]. A few deep learning recognition experiments of some of our target species (snakes and lizards) have been performed, but with image samples taken by digital or aerial cameras in labs or natural habitats, not with camera trap images similar to ours. Recently, ecologists, biologists, engineers, and volunteers have begun collaboration in various citizen science projects and supporting research by collecting wildlife data at large geographic and time scales [2]. Zooniverse, iNaturalist, Pl@ntNet, and Flora Incognita are crowd-sourced projects engaging citizen scientists [17].

Millions of volunteers help professional researchers to produce reliable and accurate data by manually reviewing, labeling, and analyzing images [17]. Due to the availability of crowd-sourced datasets and the accessibility of benchmark deep learning frameworks, more research is being published for our target taxa with gradual improvement over time. The related literature survey can be focused on two perspectives: (1) animal species identification from camera trap images using machine learning techniques and (2) target species recognition using machine learning techniques.

#### 1.2.1. Camera Trap Dataset with Machine Learning

In 2013, the first complete analysis with a camera trap dataset with machine learning was performed by Yu et al. [13], with improved sparse coding spatial pyramid matching (ScSPM) as a feature extractor and a support vector machine (SVM) as a classifier. The recognition performance achieved up to 82% accuracy [13]. The first CNN application in camera trap species identification was by Chen et al. [22]; the authors presented a comparison between the traditional bag of visual words (BOW) and a proposed DCNN. The authors extracted features with a scale-invariant feature transform algorithm in the BOW method and classified animals with a linear SVM [22]. With a very challenging, noisy dataset consisting of 14,346 training and 9530 testing images of 20 common species, DCCN (38.315% accuracy) outperformed BOW (33.507%) [22].

After 2016, additional studies have conducted experiments to identify species with pretrained DCNN architectures and publicly available citizen science datasets. Gomez et al. [23] identified 26 classes of animal species from the highly unbalanced Snapshot Serengeti dataset with eight variations of CNN frameworks AlexNet, VGGNet, GoogLeNet, and ResNets. The number of layers of the mentioned CNN architectures varied from 8 (AlexNet) to 152 (ResNet-152), where the ResNet-101 architecture achieved the best performance [23]. In another approach, Norouzzadeh et al. [15] not only detected the presence of species, but also identified further attributes, counting individuals and characterizing behaviors (the presence of young species). The authors experimented with nine independent pretrained architectures with 48 species in the 3.2 million images of the Snapshot Serengeti dataset [15]. Similar work has been found in the work of Nguyen et al. [12], where they designed a twofold task: (a) first, filtering images containing an animal from a set of Wildlife Spotter project datasets, (b) then, classifying species automatically. They implemented several pretrained CNN architectures where VGG16 achieved more than 96% in recognizing animals in images, and ResNets 50 reached close to 90% in identifying three common animal categories (bird, rat, bandicoot) [12].

In 2018, Schneider et al. [14] applied two state-of-the-art object detection algorithms, Faster Region-Convolutional Neural (FRCN) Network and You-Only-Look-Once (YOLO) v2.0, to identify and quantify animal species on two different datasets (Reconyx Camera Trap and the self-labeled Gold Standard Snapshot Serengeti). The analysis reveals that Faster R-CNN represented promising results (93% accuracy), whereas YOLO (76.7% accuracy) provided speed advantages with a real-time performance [14]. Schindler and Steinhage (2021) [24] evaluated a combined approach of Mask R-CNN and Flow-Guided Feature Aggregation in animal detection, identification, and action recognition in the video clips. They classified animals and their behavior for deer, boar, fox, and hare with mostly night vision video clips of camera traps placed in Bavaria, Germany [24].

The study by Chen et al. in 2019 [25] is the most relevant work to our research, where the authors identified six taxa (badger, bird, cat, fox, rat, and rabbit) from video camera surveillance. They trained 8368 images with two different networks; a self-trained CNN network and a pretrained model AlexNet, where AlexNet outperformed the self-trained model [25].

#### 1.2.2. Target Species Recognition Using Machine Learning

Noticeable deep learning species recognition experiments that include some of our target species (snakes and lizards) using online or aerial view datasets (but not from camera trap images) have been completed recently. Sahu [26] detected and counted lizards from drone view images applying pixel-wise image segmentation deep learning approach U-Net. The author trained the model with 600 online datasets and achieved 98.63% accuracy using a batch normalization layer in the U-Net architecture [26]. In another approach, Aota et al. [27] detected *Anolis carolinensis* using drone images acquired from the Ogasawara Islands in Japan. The trained Single Shot Multibox Detector (SSD) model detected lizards with 70% precision [27].

In 2018, Abdurrazaq et al. [28] classified five venomous snake species from Indonesia from 415 sample images using three self-trained CNN models: shallow, medium, and deep CNN architectures by changing filter size and adding more layers. With a fivefold cross-validation process, the medium architecture offered the best performance with an average accuracy of 82% [28]. Patel et al. [29] conducted a real-time identification experiment on nine species of snakes from 247 sample images from the Galápagos Islands, Ecuador, by applying an object detection and image classification approach. The authors implemented region-based convolutional neural network (R-CNN) architectures for object detection with four distinct pretrained models where ResNet achieved the best classification accuracy [29].

In a comparative snake identification study, Rajabizadeh and Rezghi [30] assessed holistic methods, k-nearest neighbors algorithm (KNN), support vector machines (SVM), logistic regression (LR), and pretrained CNN architectures. In the traditional methods, the researchers combined the dimension reduction process, principal component analysis (PCA), and linear discriminant analysis (LDA) to extract essential features where LDA performed better than PCA. However, the CNN-based pretrained model MobileNetV2 outperformed the results of holistic methods. Abayaratne et al. [31] classified six snake species from the Sri Lanka region from 2000 images using four pretrained models: InceptionV3, VGG16, ResNet50, and MobileNet, and a self-trained architecture. Among them, MobileNet yielded the best result with 90.5% accuracy. In a similar work, Progga et al. [32] categorized venomous and nonvenomous snakes from 1766 samples. The authors compared the evaluation of several pretrained models by tuning the optimizer, where fivefold cross-validating for the SGD optimizer provided the best result.

The AI-based Snake Species Identification Challenge (SnakeCLEF) has introduced a new platform for automatic snake species recognition experiments by providing labeled data with geographical information [33]. In 2020, Bloch et al. [34] used a Mask Region-based Convolutional Neural Network (Mask R-CNN) with EfficientNets to distinguish between 783 snake species from 245,185 training and 14,029 validation samples. The best model achieved a macro-averaging score of 0.594. In the 2022 SnakeCLEF challenge, Yu et al. [35] investigated EfficientNets and the transformer models in the snake classification task. The integration method achieved a macro 𝑓1 score of 71.82% [35]. In the same challenge, researchers in [36] accomplished an improved score of 82.65% by applying the ensemble approach of several pretrained and MetaFormer models.

#### 1.2.3. Objective

In this work, we attempted to build an animal recognition framework to distinguish between lizards, snakes, frogs/toads, and background in camera trap images with a DCNN approach. The experiment evaluated two frameworks: a self-trained CNN model and two pretrained models, VGG16 and ResNet50. While considering the self-trained model, we aimed not to beat the current state-of-the-art image classification architectures or pretrained models such as VGG16 or ResNet50 but to find the influence of an efficient network and augmentation parameter on a relatively small dataset. The outcomes deliver evidence of (1) the advantage of the robust network and pretrained weight on feature extraction and classification and (2) the impact of augmentation parameters for a small dataset on the three models’ performance. We hope the findings will help the wildlife research community advance more efficient ecological monitoring, especially for small animal species.

## 2. Dataset

The data material used in this study was collected from five counties across Texas, USA, between March and October 2016 [37]. The researchers of Texas A&M University conducted a camera trap experiment with 26 RECONYX PC800TM cameras to study the diversity of animal species, primarily terrestrial squamates [37]. The cameras were established maintaining at least 450 m distance from each other and mounted to a metal conduit at the height of ~2 m with the lens pointed down towards the ground [37]. Further, the cameras were deployed in conjunction with four drift fences, assuming that this technique would improve the detection of squamates by slowing and directing their movement through the camera detection area [37]. The cameras were programmed to capture images every 30 s using a time-lapse triggered mode, eventually producing thousands of images in 4352 camera-trap days [37]. However, most images were empty, with no species in them.

**Figure 1:**
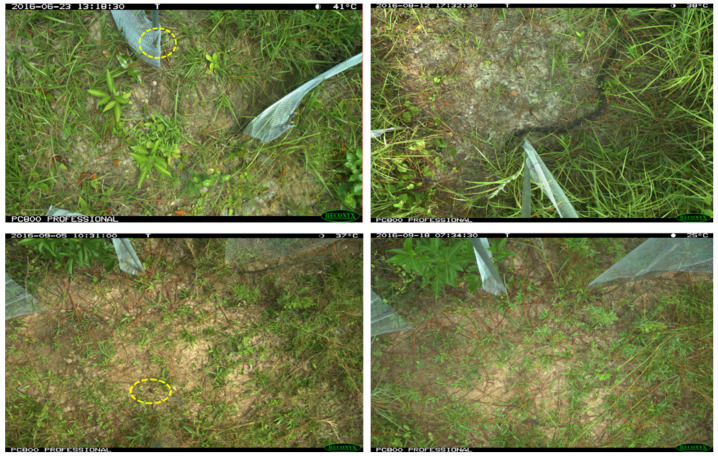
Samples of target species lizard (**top left**), snake (**top right**), frog/toad (**bottom left**), background (**bottom right**) from the camera trap dataset showing relative body size and shape. Furthermore, images have cluttered background, confusing body color with nature or not recognizing body parts hidden behind vegetation.

**Figure 2:**
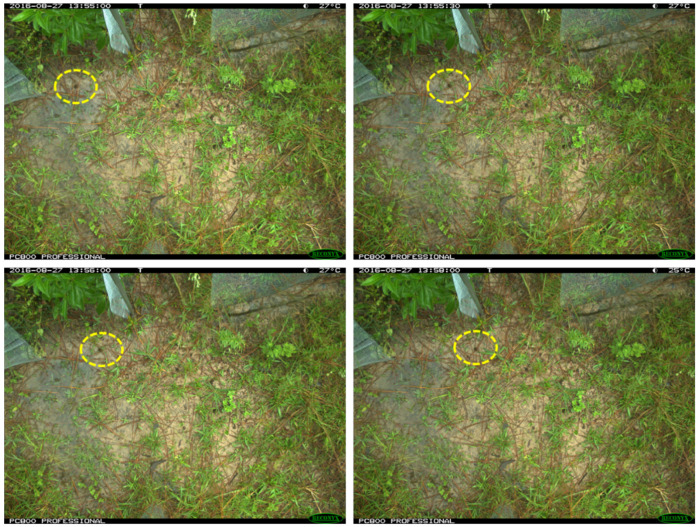
Samples of sequential frog/toad images from the camera trap dataset.

**Figure 3:**
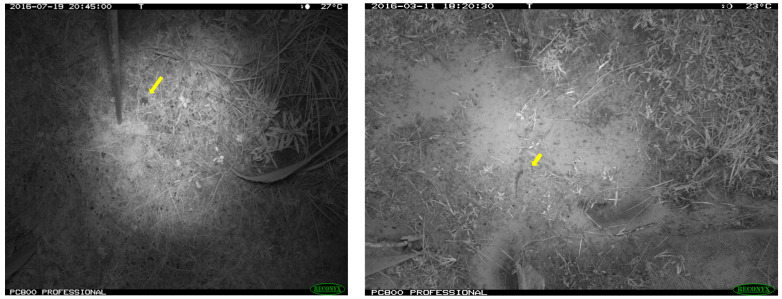
Samples of night-vision IR images of a frog/toad (**left**) and a lizard (**right**).

The experiment dataset consisted of ~700 images of eleven different snake species, ~600 images of four toad species, and ~1400 images of a single species of lizards (provided in Appendix A, Table A1), all of which were examined and labeled by the researchers of Texas A&M University. The dataset exhibited diverse and challenging attributes as follows:

- The dataset was highly imbalanced within the three groups of species. Furthermore, the samples differed considerably with alternative day/night lighting conditions and seasonal weather, variation of animal body shape, color, texture, posture, and when partially obstructed with grass or another object or cropped out of the image.
- All the captured images had a resolution of 1920 × 1080 or 2048 × 1536 pixels, where snakes comprised a 5% to 15% pixelated area in the image background. With a dynamic body posture, lizards represented 0.025–0.35% of the whole image area, while toads comprised 0.7–0.16%, having uniform body shapes (Figure 1).

- Single target groups, especially toads and lizards, were often found in consecutive images, producing a serialized image of the same species within their background. In the dataset, toads and lizards appeared in 20–150 sequential images, and snakes could be detected in 3–15 sequential images (Figure 2).

- The infrared (IR) mode produced grayscale images with less lighting intensity variation in night-vision pictures. On the other hand, daytime images added complexity to the background due to an illumination brightness alteration (Figure 3).

## 3. Methodology

### 3.1. Deep Learning Networks

We examined the convolutional neural network (CNN) as a deep learning algorithm for an image recognition framework. CNNs are mainly composed of three kinds of layers, convolutional (CONV) layers and subsampling or pooling layers, followed by one or more fully connected (FC) layers at the end [38]. CONV layers utilize a specialized kind of linear operation called “convolution” [39] between the input image pixel value and the small learnable filters called “kernels”. An RGB image is a multidimensional matrix with the width, height, and depth dimensions [16]. Kernels are added with a randomly initialized learnable value weight and operate an element-wise matrix multiplication, resulting in relevant feature maps [16,40]. Each feature map is run through the nonlinear activation function ReLU (rectified linear unit) that prevents missing any meaningful information [41] by converting negative inputs into true zero values [16]. The pooling operation decreases the size of the feature map and eventually increases the tolerance of translation invariance to some degree of image distortion [19,38]. The reduced input size decreases computational complexity and memory usage [19] and helps to reduce the overfitting tendency [19,38]. A few FC layers map all the features from the previous convolution and subsample layers [16,40,42] and prepare it for output prediction. DCNNs structure functions hierarchically where lower hidden layers formulate low-level structures (e.g., line segments, orientations, edge), intermediate layers learn abstract elements (e.g., squares, circles), and the higher hidden layers and the output layer combine these discriminatory characteristics and provide a probability distribution within the assigned class labels [16].

Due to sparse connectivity, parameter sharing (also known as weight sharing) [38], and equivariant representations properties [39], CNNs are efficient techniques for image processing. In a CNN network, usually, smaller-sized kernels scoop small, meaningful features (edges, corners) from a larger input image [4,39]. This sparse connection process reduces the number of parameters and memory requirements, decreases the computational operation, and improves the model’s efficiency [39]. Furthermore, the CNN architecture applies multiple filters to its inputs with the same weight to extract salient features [39]. This weight-sharing process reduces the number of parameters, improves its generalization ability [39,43], and acts as a shift-invariant [40].

We developed and tested three CNN frameworks for this study, a self-trained framework (CNN-1) and two transfer learning frameworks with pretrained models VGG16 and ResNet50. While training the networks, the image augmentation and trainable parameters were selected in a particular manner to achieve the best accuracy of each model. As mentioned earlier, our goal was to explore the impact of varying design configurations and parameters on the model’s behavior and level of performance.

#### 3.1.1. Self-Trained Model

This model comprised four sets of CONV layers, where each set was assembled with a sequence of CONV, activation, batch normalization, pooling, and dropout layers, respectively. The batch normalization and dropout layers were added to regularize the model and improve efficiency. All the 2D feature maps were flattened into a one-dimensional vector at the end of the CONV layer block. Two dense layers were added along with the dropout layer as an FC layer with a SoftMax activation layer to predict classes.

**Figure 4:**
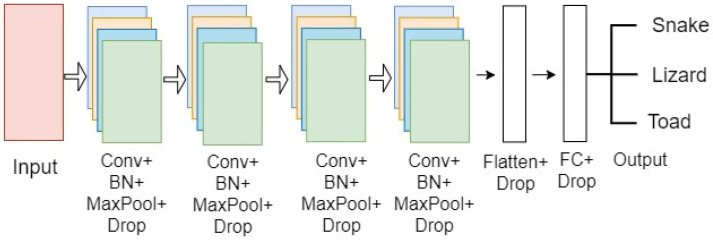
An example of a four-layer CONV architecture for the snake, lizard, and toad classification.

The number of filters in the first two layers was 32, and in the final two layers—64, with a kernel size of 3 × 3 and a pool size of 2 × 2. Stride was kept at the default value of one, and the padding size was chosen as valid padding for the final architecture. Figure 4 illustrates the CNN model’s configuration.

#### 3.1.2. VGG16 Model

**Figure 5:**
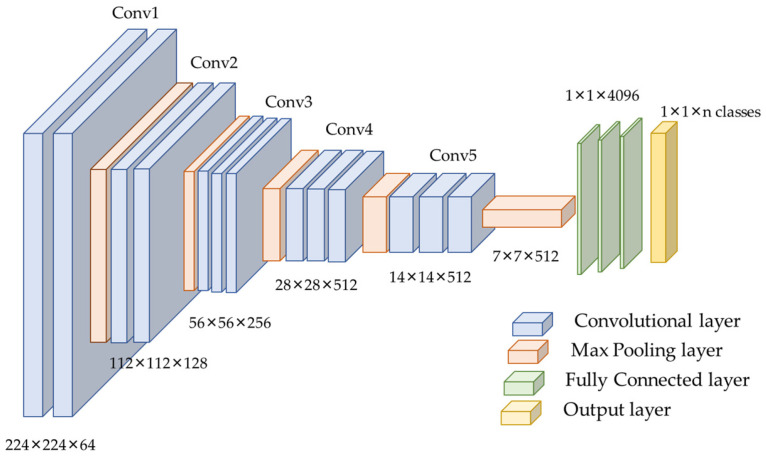
VGG16 architecture used for this experiment.

Considering the data size and class similarity with the ImageNet dataset, we utilized the VGG16 model for training our samples. VGG16 is a CNN model introduced by K. Simonyan and A. Zisserman in 2014 [44]. The sixteen CONV layers define the depth of the network. The network is considered more straightforward than other benchmark networks as it comprises 3 × 3 CONV filters [16,29]. The max-pooling was performed over a 2 × 2 pixel window, with the stride size set to two [44,45]. The original network consists of two FC layers, each with 4096 nodes, followed by a SoftMax classifier of 1000 neurons [44]. VGGNet is computationally expensive as it contains 138 million parameters [44]. Figure 5 depicts the VGG16 model’s configuration.

#### 3.1.3. ResNet50 Model

The residual neural network, commonly known as ResNet, was first proposed by He et al. in their research work [46] in 2015. This extremely deep network exhibits powerful performance and fine convergence attributes [47]. Researchers have found that the deeper network suffers the vanishing gradient problem where the accuracy gets saturated and eventually degrades with an increasing network depth [46]. This issue creates an obstacle in the convergence and shows an overfitting tendency by increasing the training error [46]. Residual neural networks utilize skip connections (a shortcut connection to jump over some layers) that allow the information to flow from the input or the earlier layers into deeper layers to offset the vanishing gradient problem [46]. Skip connection also works as identity mapping adding the input from the previous layer directly to the output of the other layer. The idea is that a deeper network performs better, or at least the same, as shallower networks.

**Figure 6:**
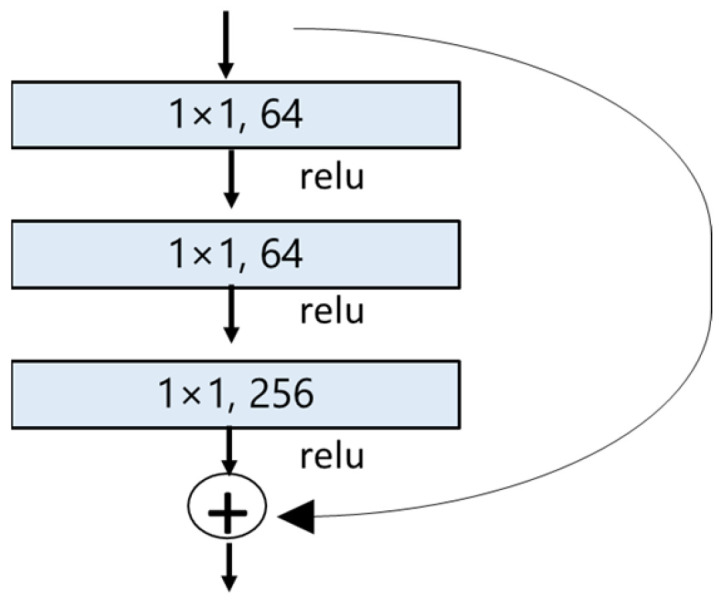
BottleNeck building block for ResNet50 [46]. The shortcut connection (skip connection) performs identity mapping.

In our project, we utilized ResNet50, which contains 50 neural network layers with the BottleNeck design. The BottleNeck block uses three CONV layers, 1 × 1, 3 × 3, and 1 × 1 CONV layers, as seen in Figure 6 [46,48,49]. All it does is use a 1 × 1 convolution operation to reduce the dimension (channel) of the input, again performing an expensive 3 × 3 convolution, then using another 1 × 1 to project it back into the original shape [46,48,49]. The 3 × 3 layer is a bottleneck with smaller input/output dimensions done by the 1 × 1 layers [48,49].

### 3.2. Image Classification Framework

**Figure 7:**
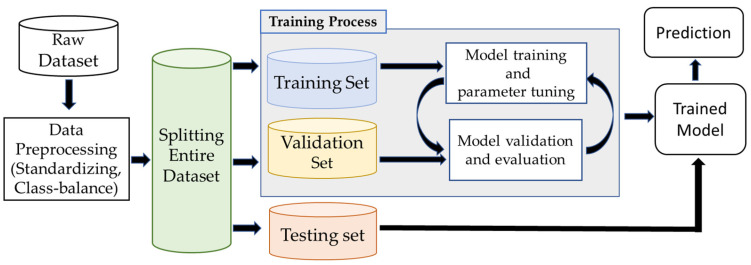
The workflow of the image classification pipeline involves preprocessing the raw dataset, training and validating architecture, and testing the final model with different sets of samples.

The image classification infrastructure involves preprocessing the raw dataset, training, validating the deep learning architecture, and evaluating the final model with unseen test data, as shown in Figure 7. Available data are divided into training, validation, and a test set. The CNN model learns salient features during training and extracts relevant information from the training data. Here, each datapoint (image) was trained with its corresponding category (i.e., snakes, lizards, toads) as labeled by the researchers originally using the Texas A&M University camera trap dataset. The validation dataset monitored the performance and tuned the hyperparameter to make the best fit of the model. The model measured the difference between the actual and predicted labels in forwarding propagation. In the backpropagation operation, the model updated the learnable parameters (weight, bias) to fit the best model for the given dataset.

The model was iterated through several training operations by changing parameters such as model layers, filter size, learning rate, epoch size, and batch size. After gaining satisfactory accuracy through several training and validating processes, the best-performing model was chosen as the final model and evaluated with unseen test data.

### 3.3. Data Preprocessing

Our experiment endured (1) a significantly small dataset for the image classification problem, (2) highly imbalanced samples for all the animal classes, and (3) unstandardized input as the images varied in resolution and scale. Efficient computation of deep learning networks required careful consideration to minimize the above difficulties. The original camera trap dataset contained 700 snake images, 600 toad images, and 1400 lizard images. Before feeding the data into the code, we applied an oversampling technique that increased the snake and toad samples toward equalizing the imbalanced dataset. We rotated snake and toad images at 180-degree and 90-degree angles of the original images and added the transformed samples to the existing directory. Eventually, this process led to expanding the overall number of samples in the dataset, as shown in Table 1.

The data division for training, validation, and testing was developed randomly from the whole dataset to ensure that the training dataset included all the variations described above, having different day/night images, changing weather conditions, and background. A sequence of images was indiscriminately put into the training, validation, and test subsets to achieve this randomness for most cases.

### 3.4. Augmentation

We applied a wide range of augmentation techniques to transform the original images of the training dataset using the data generator function by Keras [50]. This function modifies training samples in various forms and shapes [15] and replaces the original dataset in the training phase [15,51]. Random transformations such as zooming, shearing, and rotating strengthen the robustness of architecture by intuitively adding variations in object pose and location of the samples [52]. The aim was to increase the model’s generalization ability to predict an unseen image of the target classes subsequently. Data augmentation often helps to reduce training and validation errors, thus helping to minimize overfitting tendencies [50].

**Figure 8:**
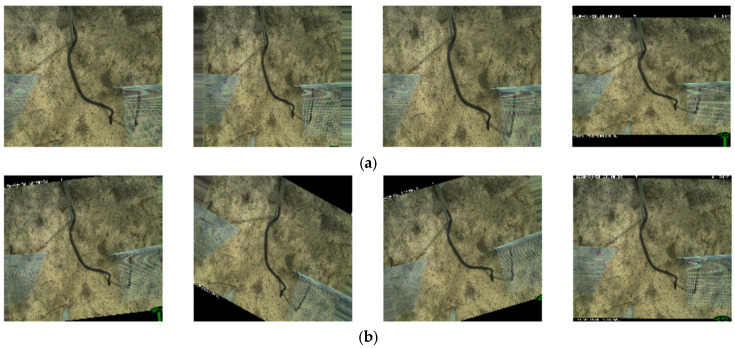
(**a**) Example of a zooming operation with a range of 30% where the first three images on the left are zoomed in, and the fourth image is zoomed out. (**b**) Random image rotation between 0 degrees and 30 degrees.

This method augments samples using different transformation techniques with a given range or boundary value. For example, Figure 8 depicts rotation augmentation with a range of 30%, where the image randomly rotated between 0-degree to 30-degree angles. Similarly, the zooming operation was configured with the zoom range (1− range, 1+ range) argument applying zoom-in and zoom-out transformation [53]. In Figure 8a, we can see that with a specified 30% zoom range, the first three random images became closer while the objects in the fourth image went farther away between the 70% (zoom-in) and 130% (zoom-out) boundary values.

The augmentation parameters and range were selected for this work considering the complexity of the target species’ attributes, image quality, and model architecture. After several trials, the augmentation parameters in Table 2 were chosen for the final model.

### 3.5. Evaluation Metrics

A model’s performance can be evaluated by how the classifier recognizes new or unseen data from the same distribution of samples used to train the model. After training the model, we validated the performance with 250 images of each group. The training and validation curve (learning curve) indicates the model’s performance over the computation. To measure the generalization ability of the CNN classifier, we evaluated the model with a separate set of test data of 100 images from each group. The test result was calculated with some statistical metrics such as accuracy, precision, recall, and F1-score based on true positives (TP), false negatives (FN), false positives (FP), and true negatives (TN). Here, TP and TN represent the number of correctly identified target species (snakes, toads, lizards) among the given samples, while FP and FN denote misclassified classes of species [15,54].

Accuracy scores tell how often the models produced correct results from the dataset:

(1) $$\mathrm{Accuracy}=\frac{\mathrm{True}\;\mathrm{Positive}\;\left(\mathrm{TP}\right)+\mathrm{True}\;\mathrm{Negative}\;\left(\mathrm{TN}\right)}{\mathrm{Total}\;\mathrm{number}\;\mathrm{of}\;\mathrm{data}\;\mathrm{samples}}$$

The precision score determines the ratio of correctly identified target group images to all the images predicted as that particulargroup:

(2) $$\mathrm{Precision}=\frac{\mathrm{True}\;\mathrm{Positive}\;\left(\mathrm{TP}\right)}{\mathrm{True}\;\mathrm{Positive}\;\left(\mathrm{TP}\right)+\mathrm{False}\;\mathrm{Positive}\;\left(\mathrm{FP}\right)}$$

Recall calculates the ratio of correctly identified target group images to all the images of that target group in the test data:

(3) $$\mathrm{Recall}=\frac{\mathrm{True}\;\mathrm{Positive}\;\left(\mathrm{TP}\right)}{\mathrm{True}\;\mathrm{Positive}\;\left(\mathrm{TP}\right)+\mathrm{True}\;\mathrm{Negative}\;\left(\mathrm{FN}\right)}$$

F1-score represents a weighted average of precision and recall:

(4) $$F1=2\times \frac{\left(\mathrm{Precision}\times \mathrm{Recall}\right)}{\left(\mathrm{Precision}+\mathrm{Recall}\right)}$$

## 4. Experiment and Results

We designed a training scheme of CNN-1 and the pretrained models considering the target species’ physical attributes, sample size, species community, and the desired experiment outcome. The dataset had three distinctive target groups where snake species have larger body sizes (5% to 15% of the pixelated area in the background) than either lizards or toads (0.025–0.35% of the pixelated area). Consequently, snake species might have an unfair advantage while training and classifying three groups. Therefore, we conducted binary experiments for each target group with empty camera trap images referred to as background. These images were from the exact location and environment but contained none of the target groups. The binary experiments aimed to understand how the model can differentiate between samples with target species from an empty image. Finally, we conducted a multiclass experiment with all four classes (three target species and background) and compared the recognition performance. Here, we highlight the essential experiments (source code link is given in Supplementary Materials) and discuss the corresponding results from the camera trap species classification analysis of both CNN-1 and the pretrained models.

### 4.1. CNN-1

The self-trained model was optimized by adjusting several hyperparameters such as learning rate, dropout percentage, batch size, number of epochs, and other parameters. The dropout layer with a 25% value after the CONV and dense layers showed a favorable outcome for tuning. The architecture was compiled with the categorical cross-entropy loss function and the Adam optimizer with a learning rate of 0.01. With an input image size of 150/150 and a batch size of 32, all the computations were executed for 100 epochs.

The training phase of CNN-1 was incorporated with numerous augmentation techniques such as zooming, shearing, rotating, flipping, and horizontal shift with varying parameters and ranges. Several experiments found that the CNN-1 model started performing worse after introducing the image augmentation process. With a low transformation range, the accuracy and loss curves started showing fluctuation, and with the increasing amount of augmentation parameters, the learning curve suffered from an overfitting trend. Furthermore, the evaluation performance started decreasing after introducing the augmented dataset in the training period.

**Figure 9:**
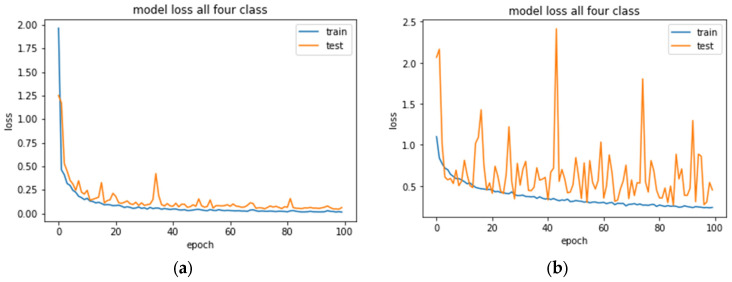
Training and validation loss curve of CNN-1 for the multiclassification problem for 100 epochs. (**a**) No augmentation was implemented. (**b**) After applying the augmentation parameter.

The loss plot in Figure 9a depicts a smooth convergence with a training curve with no sign of overfitting. On the other hand, Figure 9b shows that the validation loss curve featured fluctuating movements around the training loss, indicating that the model struggled to recognize species in the validation set. One of the reasons behind the oscillation might be the data augmentation method, where the model was trained with a batch of a slightly modified version of samples in each epoch. As the model was already trained with fewer images, the transformed images hindered the training process for the CNN-1 model. Eventually, the validation dataset did not provide sufficient information to evaluate the model’s generalization ability.

However, we did not want the model to overlearn from training with a limited sample size, where the model would memorize the training data and perform poorly after being deployed in production. To improve the model’s generalization ability, we trained the model with a minimal augmentation range or parameters, as listed in Table 2. Table 3 summarizes the results of the binary and multiclassification experiments.

As seen in the accuracy column, the self-trained binary species classification on camera trap images achieved good performance, where the toad group had a higher value (96%) than the snake (83%) or lizard groups (77%). The multiclassification results yielded lower accuracy, 72%, than binary classifications since added classes bring more complications within the network due to the additional characteristics of the new samples. The F1-score for the multiclass problem follows a similar trend where the models could recognize toads better than the other two species. We also see that CNN-1 needed help differentiating between lizards and background samples. Background images were misclassified as lizard images in the binary and multiclassification models.

### 4.2. Pretrained Models

**Figure 10:**
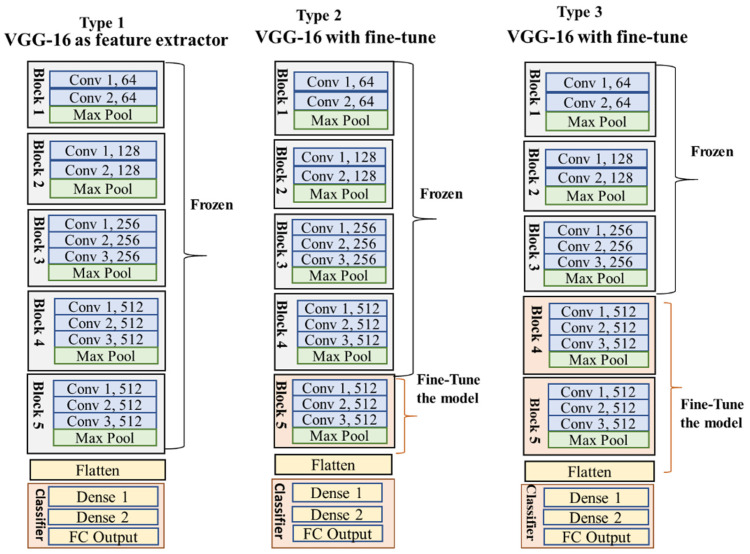
Type 1, Type 2, and Type 3 are VGG16 models. Type 1 is a feature extractor model with frozen CNN blocks and used previous features. Type 2 and Type 3 are fine-tuned models that retrained the samples with a few CNN blocks to find intricate features from our data.

**Figure 11:**
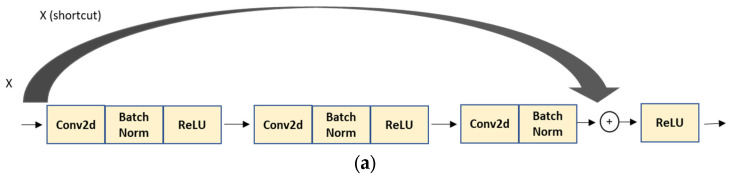

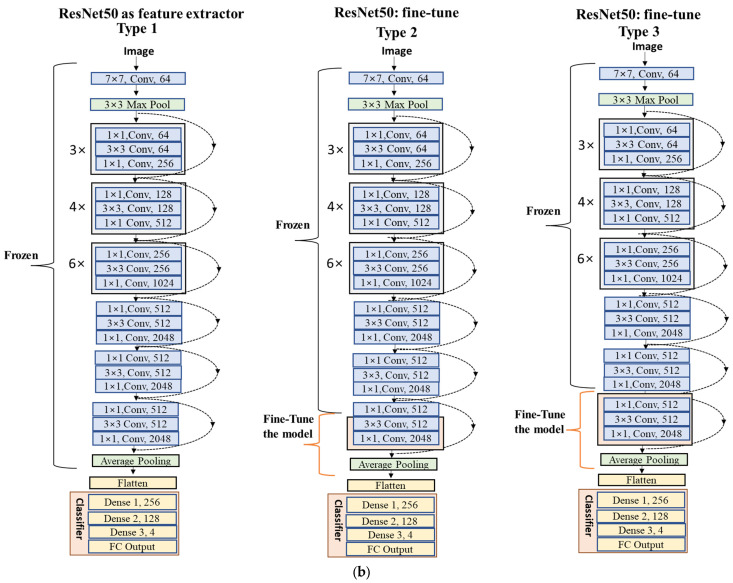
(**a**) Bottleneck residual module structure of each CNN block. (**b**) Type 1, Type 2, and Type 3 are ResNet50 models for multiclassification models. Type 1 is a feature extractor model and Type 2 and Type 3 are fine-tuned models.

The pretrained VGG16 and ResNet50 were already trained with millions of images of the ImageNet dataset, including our target groups. Therefore, we trained the model with an ImageNet weight to benefit from learned features across different objects and groups [20,42]. However, our data were quite challenging compared to the ImageNet dataset as the camera trap samples consisted of cluttered backgrounds, confusing body color with nature, hiding body parts behind vegetation or fencing, and varying lighting conditions. That is why we utilized the model for both feature extraction (referred to as Type 1) and fine-tuning (named Type 2 and Type 3) through several experiments to obtain the optimal output (Figure 10 and Figure 11).

Type 1 for both VGG16 and ResNet50 (Figure 10 and Figure 11) modified only the classifier containing the FC and output layers while maintaining the original base model (series of CONV and pooling layers). This technique allowed us to utilize the previously learned patterns and representations of the ImageNet sample. The new classifier was adjusted with new dense and SoftMax layers. The added FC and output layers were retrained, and the actual base model combined the representations of camera trap images as the output. However, the Type 1 model needed to provide satisfactory outcomes for both architectures, as shown in Table 4, Table 5, Table 6 and Table 7. The results indicate that the model needed to be trained with an adjusted CNN layer and a pooling operation, commonly known as fine-tuning, to extract salient and specific features to represent our data better.

Fine-tuning comprised unfreezing a few of the top layers of the pretrained model and jointly training both the newly added classifier and these top CNN blocks. This technique was influenced by the observation that the early layers of the CNN network produced highly generic features such as visual edges, colors, and textures from ImageNet [42,55]. Therefore, keeping the bottom layer frozen allowed us to reuse the formerly learned local features for our task [20,42]. At the same time, training the top CONV and pooling layers helped to obtain more abstract representations of the sample to make them more relevant to the target dataset.

#### 4.2.1. VGG16

For VGG16, we trained the network with varying CNN blocks. Here, Type 1 was used as a classifier, and both Type 2 and Type 3 models utilized fine-tuning processes, as shown in Figure 10. The classifier design was the same for all the models, an FC layer with 256 nodes and a SoftMax layer. In the training stage, the choice of an optimizer was a stochastic gradient descent with a learning rate of 0.0001 and a momentum of 0.9. Models were trained for 100 epochs with a batch size of 32. The images were reshaped to 224 × 224 per the network’s original VGGNet input configuration. The training process includes several augmentation techniques by Keras, as shown in Table 2. Table 4 and Table 5 summarize the results on a test dataset of 100 samples of each group.

The accuracy and F1-score results in Table 4 and Table 5 confirm that the fine-tuning experiments with the camera trap dataset performed better than the model trained with the adapted classifier. Though Type 1 acquired decent accuracy on the test data, the model suffered from overfitting issues on the accuracy and loss curves. Moreover, Type 3 also showed overfitting issues we observed through the learning curve while training the models. For VGG16, Type 2 provided the best performance delivering a good model fitting on the training and validation data and good generalization accuracy on the test data. The multiclass model achieved 87% test accuracy, and the binary models achieved a test accuracy of 95% for snakes, 98% for toads, and 82% for lizards against background images.

Similar to CNN-1, the VGG16 models recognized toads better than the other two groups and faced difficulty with lizard recognition. All the models adequately recognized snake species, given the unique features available from the camera trap images. The evidence demonstrates the ability of the ConvNets architecture to deal with the species classification problem and shows how fine-tuning specializes the network on the camera trap groups.

#### 4.2.2. ResNet50

Similar to the VGG16 network, we conducted three experiments with ResNet50. For Type 1, we fixed the weights and the CONV block of the architecture, whereas the Type 2 and Type 3 models were retrained with two sets and three sets of top CONV layers, respectively. All three models contained an adjusted classifier by adding two dense layers and one SoftMax layer. Network training was performed in batches of 32 using the Adam optimizer with a learning rate of 0.0001. The data were trained for 100 epochs with an input size of 224 × 224 × 3. We implemented an augmentation parameter (given in Table 2).

**Figure 12:**
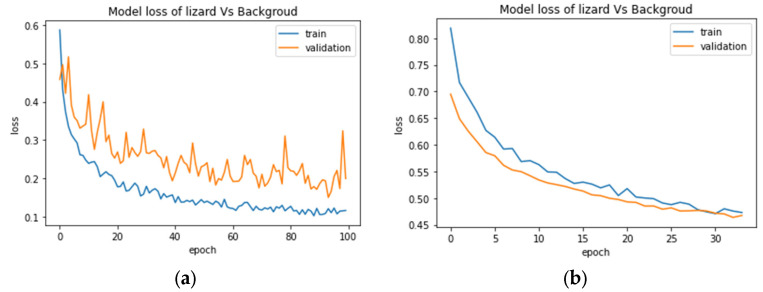
Training and validation loss curves of ResNet Type 2 for the lizards vs. background classification experiment for 100 epochs (**a**) without an early stopping, (**b**) after applying an early stopping where the operation halt at epoch 30.

In the training phase, the learning curve of training and validation of the multiclassification model and the binary classification for snakes did not demonstrate any divergence for any of the three types of architecture, which indicates no sign of overfitting. However, the binary classification for toads showed a slight tendency of overfitting only for Type 1 as the validation accuracy and loss was slightly higher than the training accuracy and loss value. However, Type 2 and Type 3 for the toad classification were not subject to overfitting and demonstrated promising accuracy. Surprisingly, the binary architecture for lizards and background experienced high overfitting issues, obtaining a low score (Figure 12a).

To counter the overfitting issue with the lizard–background binary classification problem, we started fine-tuning the architecture separately by changing layers and neurons, then adjusted the low augmentation parameter to see the impact on performance. Finally, changing the optimizer from Adam to SGD demonstrated an interesting result where the learning curve converged within 25–35 epochs and again started to divergent. To stop degrading the result after certain epochs, we implemented an early stopping function to halt the training process further and restore the best weights. Early stopping is a regularization technique that monitors the model’s performance for a given set of operations or epochs. It terminates the training process when the model stops improving its accuracy or loss value [56]. After applying this function with a patience value of three, the training stopped at 27–35 epochs and improved performance with an acceptable learning curve, and the results are shown in Figure 12b. The loss curves in Figure 12 demonstrate the impact of early stopping, and Table 6 and Table 7 list the accuracy and F1-score results for ResNet50.

Table 6 and Table 7 determine that Type 3 architecture performed better than the other two networks for multiclassification, having 86% accuracy. We consider Type 2 the best method for binary problems because those models provided better learning curves than Type 1 and Type 3. The binary classification for snakes acquired 95% accuracy, for toads—99%, and for lizards—78% (after applying the early stopping function). ResNet50 also exhibited a similar trend where the model obtained promising performance in identifying toads and snakes; however, it struggled to classify lizards from the background.

### 4.3. Performance Summary

We evaluated the performance of the classification based on the accuracy and F1-scores of three different CNN architectures on 700 snake, 600 toads, and 1400 lizard images. Overall, all the models attained very high accuracy for the binary classification of snakes (83% for CNN-1 and 96% for the pretrained models) and toads (96% for CNN-1 and 99% for the pretrained models) and achieved moderate results for lizards (77% for CNN-1 and 82% for the pretrained models). For multiclassification, the best accuracy was 87%, with VGG16 and ResNet50 yielding a marginally lower output of 86%. These results confirmed that pretrained architectures with fine-tuning would better categorize our species.

The literature review suggests the model’s performance may vary widely between dataset features and AI platforms. Chen et al. [25] classified six species with two architecture variations where AlexNet outperformed a self-trained CNN-1 model scoring 90% accuracy. In another research, Nguyen et al. [12] identified three common species (birds, rats, and bandicoots) from the Wildlife Spotter project dataset with three different transfer learning models and acquired the best result with ResNet50 (90.4%). Similarly, Willi et al. [57] obtained species identification accuracy between 88.7% and 92.7% using ResNet18 from four citizen science project datasets. While previous works revealed high classification accuracy, we have to be aware that most assessments are reported with larger species (mammals and birds) or trained with greater volume of data.

## 5. Discussion

### 5.1. Reflection of Performance

Our work leveraged the VGG16 and ResNet50 architectures with varying CNN blocks and various augmentation effects. The fine-tuning approach indicates that training the transfer learning models with the last few unfrozen blocks of CNN layers (Type 2 and Type 3) and a modified classifier attained the highest recognition accuracy. We also demonstrated the successful implementation of a self-trained model with varying augmentation properties, allowing us to train a model with a wide range of form- and shape-modified objects. However, CNN-1 exhibited low performance with high augmentation parameters while recognizing species from images. The finding provides several valuable insights for the automated recognition of animal species, as discussed below.

1. The experimental outcome reveals that augmentation parameters play a vital role in learning accuracy. The two kinds of models, self-trained (CNN-1) and transfer learning (VGG16 and ResNet50), react differently to augmented images, while pretrained models show more tolerance to transformed images. Without applying augmentation, CNN-1 provided remarkable training, validation, and testing accuracy for the given samples with no overfitting issues (Figure 9a). On the other hand, we observed a large generalization gap in the learning curve while training with a high augmentation threshold (Figure 9b).
  In ideal cases, augmentation is supposed to improve the model’s generalization ability and enhance the model’s performance. However, we noticed an opposite effect of the augmentation process, where a high augmentation parameter deteriorated the performance of CNN-1. The reason was likely that CNN-1 suffered from data deficiency. Due to the augmentation procedure, the model was trained with a new set of slightly modified data in each epoch. This made the model inefficient in learning the individual meaningful pattern of the target object. Another point that must be mentioned is that the self-trained model was not robust enough to train on the small challenging dataset. The high augmentation parameter added extra difficulty to the training process, negatively impacting the overall evaluation task of CNN-1. This obstacle can be overcome by training the model with a large amount of data, where the model will get sufficient information to learn salient features and improve the recognition ability. Moreover, there is always room for improvement in the deep learning architecture and tuning the model with the proper hyperparameters to optimize the performance.
  Nevertheless, a good learning curve with high training and validation accuracy (Figure 9, left) attained without applying augmentation can be misleading because the CNN model might memorize the data instead of learning the target features from the pixel value. Training a model with various image-augmented samples can aid the model in learning features invariant to transforms, such as left-to-right or top-to-bottom ordering [58]. The augmented data will thus represent a more comprehensive set of possible datapoints, eventually preventing the network from simply memorizing the samples. Keeping this in mind, the model was trained with considerably lower augmentation parameters than the value given in Table 2.
  As per the above analysis, deep learning networks require a large amount of training data for good accuracy, even if we apply the augmentation process [22]. Moreover, CNN-1 could identify target species from a similar location and background environment to those used while training the model. The accuracy might decrease drastically if we test the model with images taken from different locations. Even the VGG16 or ResNet50 models might need help recognizing classes if combined with different surroundings or were never introduced during training. That is why we suggest that all the automated ecological recognition outputs from the model should only be relied upon with human supervision, primarily for small-bodied species [59,60,61,62].
2. We also observed that the toad classification performed comparatively better, while many background samples were misclassified as lizards. The possible reason was that the original 600 toad samples were augmented by rotating 180 degrees and added to the existing dataset, producing repeated versions of the same image. Furthermore, most toad images consisted of night-vision samples that benefitted from blurred background features and provided a better view of the target object (Figure 3). Due to less complexity and disparity within the samples, the learning curve and test accuracy for toad classification showed optimum results. At the same time, lizards have a small body and a color that resembles the background properties, making feature extraction more complex during the training of models, which is likely why it was hard to distinguish the lizard images from the background images.
  The results also depict misclassifications between the snake and background images in all three models. Eleven snake species were in the snake group (Table A1), compared to a single lizard species and four frog/toad species. Thus, the snake dataset contained more body shape and size variations within the 700 sample images than the lizard and frog/toad groups. Most importantly, the complexity of background colors, vegetation, and shadows makes snakes harder to recognize through unique features and harder to differentiate from the background.

### 5.2. Limitations and Recommendations

1. Though the models provided very good precision and accuracy, the question arises about the reliability of classification methods working with camera trap images [57,60,61], especially with small-bodied species (lizards and toads in our case). One of the limitations of the image classification technique is the process of labeling training samples, where the whole image is annotated as an object [20]. As this process does not provide the specific focus part of the image, the model learns the background information as a feature to represent the target species [20]. Therefore, whether the network considers the correct areas as a target while training remains uncertain; this problem worsens when we train the architecture with a small dataset where the model might become biased to any particular entity in the background and is erroneously considered an essential feature.
  Additionally, pretrained and self-trained architectures downsampled the raw data into 224 × 224 and 150 × 150 consecutive images, as training the models with the original images (1920 × 1080 and 2048 × 1536 pixels) was computationally expensive. Resolution reduction speeds up the data processing [63] and decreases the memory requirement while training a deep CNN [64]. However, the downsizing process can lose important details [63] for a smaller-bodied animal in a complex background [64].
  We suggest that automatic identification through a classification process with high accuracy should be considered with skepticism for small species in an ecological environment [57,60,61]. From a technical viewpoint, training a model with a large dataset will mitigate the problem. Furthermore, an alternative labeling method can be investigated to better teach models about the complex features of species within a challenging sample set.
2. While dividing the dataset, Norouzzadeh et al. [15] did not put similar images in their training and testing sets. The motivation was that overly similar images in the training and testing sets provide exemplary performance in the model’s evaluation but might have a poor generalization ability for unseen data as the model can memorize the samples in the training phase [15]. However, the researchers experimented with over 3 million images, where they trained the model with 284,000 samples and separated 3800 datapoints as a test set [15]. In our work, we did not have the luxury to put a sequence of toad or lizard samples only in the training or testing set due to the available data limitation. Though our model achieved high accuracy, it comprehended similar or somewhat repetitive images due to sequential attributes. Therefore, the trained model will likely have lower performance while predicting species from a captured event from a new location [57,62].
3. When reusing the trained models to identify diversified species or animals from a different territory, we have to consider the experimental and resource limitations. The models inherit some degree of bias as the dataset covers examples from specific taxa and geographic habitat. Additionally, as reported earlier, a big portion of the training and testing sets contain a series of similar toad and lizard images. Though the models delivered high performance with our data, it is impractical to assume that these models will act well with untrained images from a different ecological project. For future research, the study should extend by including species from different taxa and regions. Furthermore, we recommend that AI-based models be retrained with updated data and undergo continuous development.
4. New computer vision complex networks that can discriminate the background patterns and recurrent motions of “external” objects introduced in frames need to be investigated. Other deep learning models can be explored to seek better recognition solutions, such as MobileNet, EfficientNet, Ensemble learning [65], MetaFormer models, transformers with encoding layers, etc. The potential of object detection models such as you-only-look-once (YOLO) or region-based convolutional neural networks (RCNN) needs to be studied to facilitate identifying the location of a target object of interest in an image and then classify them with a label. A significant amount of image data, a better labeling procedure, and a robust network can minimize the misclassification by providing good recognition accuracy.

## 6. Conclusions

This study explores for the first time an AI-based automated classification solution for herpetofaunal groups; snake, lizard, and toad images acquired from camera trap project. In this paper, we evaluated three independent deep learning models, including a customized CNN model, for species classification. The results demonstrate a satisfactory level of performance for deep learning models with high training, validation, and testing accuracy in each phase and show the possibility of successfully identifying snakes, lizards, and toads. For the self-trained model, the experiments were conducted by gradually increasing various factors: augmentation parameters, network complexity, and the number of epochs to investigate their effect on the performance. For CNN-1, we found a direct relationship between model accuracy and the augmentation range for network training. We noticed that low augmentation parameters generated less variation in the dataset, which caused an increment in the performance as the model analyzed similar features at each epoch. Similarly, a higher image transformation range degraded the accuracy as the model could not learn the features properly from the small target samples. The findings validate that the CNN-1 architecture will be able to recognize the target species if the quality and the background view of the image belong to a similar circumstance to those provided for this project.

In conclusion, promising levels of accuracy can be achieved by increasing the sample size, building a robust model, and fine-tuning hyperparameters of models for the species classification task. On the other hand, the pretrained models VGG16 and ResNet50 showed high performance in recognizing species as the architectures could incorporate the knowledge previously gained from other images into our species group. This paper proposes a strategy for better implementation by unfreezing some top layers, finetuning, and re-training the target species. The performance of both models was comparable as they share a similar framework. ResNet50 achieved 86% accuracy for classifying all three species groups but suffered overfitting issues in recognizing lizards in binary classification problems. Overall, VGG16 attained the best performance with 87% accuracy for multiclassification, good generalization accuracy on the test data, and no sign of overfitting issues. Further investigation should be continued to explore advanced deep learning networks, find optimal parameters, and collect more sample data to obtain an automated species recognition solution.

## Figures and Tables

**Table 1:** Balanced dataset partitioning for training, validation, and test subsets of camera trap images.

Camera Trap Data Subsets for Model Testing				
	Snakes	Lizards	Toads	Background
Training	1250	1250	1250	1250
Validation	250	250	250	250
Test	100	100	100	100

**Table 2:** Augmentation parameters for the CNN-1,VGG16 and ResNet50 models.

Augmentation Techniques	Parameters and Range for CNN-1	Parameters and Range for VGG16 and ResNet50
Rotation	10%	30%
Flipping	0.1	0.2
Shearing	0.1	0.2
Horizontal shift	True	True
Zooming	10%	30%
Fill mode	Nearest	Nearest

**Table 3:** Classification results of CNN-1 with 100 test samples of each species.

	Multiclassification				Binary Classification					
	All Four Classes				Snakes vs. Background		Toads vs. Background		Lizards vs. Background	
	Snakes	Toads	Lizards	Background	Snakes	Background	Toads	Background	Lizards	Background
F1-score	0.80	0.82	0.76	0.53	0.82	0.84	0.96	0.96	0.72	0.80
**Accuracy**	**72%**				**83%**		**96%**		**77%**	

**Table 4:** Results of VGG16 multiclassification with 100 test samples of each species.

Multiclassification Experiment						
	**Type 1**		**Type 2**		**Type 3**	
	**F1-score**	**Accuracy**	**F1-score**	**Accuracy**	**F1-score**	**Accuracy**
Snakes	0.82	85%	0.81	87%	0.82	87%
Lizards	0.92		0.94		0.90	
Toads	0.96		0.98		0.95	
Background	0.72		0.75		0.80	

**Table 5:** Results of VGG16 binary classification with 100 test samples of each species.

Binary Classification Experiment						
	**Type 1**		**Type 2**		**Type 3**	
	**F1-score**	**Accuracy**	**F1-score**	**Accuracy**	**F1-score**	**Accuracy**
Snakes	0.85	85%	0.95	95%	0.99	96%
Background	0.85		0.95		0.94	
Toads	0.94	94%	0.99	98%	0.99	99%
Background	0.94		0.98		0.99	
Lizards	0.82	81%	0.83	82%	0.79	81%
Background	0.78		0.81		0.83	

**Table 6:** Results of ResNet50 multiclassification with 100 test samples of each species.

Multiclassification Experiment						
	**Type 1**		**Type 2**		**Type 3**	
	**F1-score**	**Accuracy**	**F1-score**	**Accuracy**	**F1-score**	**Accuracy**
Snakes	89	83%	94	85%	95	86%
Lizards	78		80		79	
Toads	96		95		99	
Background	66		71		70	

**Table 7:** Results of ResNet50 binary classification with 100 test samples of each species.

Binary Classification Experiment						
	**Type 1**		**Type 2**		**Type 3**	
	**F1-score**	**Accuracy**	**F1-score**	**Accuracy**	**F1-score**	**Accuracy**
Snakes	88	87%	96	95%	97	96%
Background	86		95		96	
Toads	91	92%	1	99%	99	99%
Background	92		99		99	
Lizards	60	66%	78	78%	80	78%
Background	70		78		76	

## Data Availability

The experiment imagery data are available at: https://doi.org/10.6084/m9.figshare.20035181.v1 (accessed on 16 April 2023).

## Supplementary Materials

The source code of the experiment is available at: https://github.com/sazidabintaislam/Camera_trap_Journal_2022 (accessed on 16 April 2023).

## Appendix A

**Table A1:** Camera trap data of toads/frogs, lizards, and snakes (various taxa), including data quantity for each target group.

Target Species	Scientific Name	Common Name	Approximate Number of Animal Images
Toads/frogs	*Bufonidae*		600
	*Incilius nebulifer*	Gulf Coast toad	
	*Ranidae*		
	*Lithobates sphenocephala*	Leopard frog	
Lizards	*Anolis carolinensis*	Green anole	1400
Snakes	*Virginia striatula*	Rough earth snake	700
	*Micrurus tener*	Coral snake	
	*Masticophis flagellum*	Coachwhip	
	*Pantherophis obsoletus*	Black rat snake	
	*Storeria dekayi*	DeKay’s ground snake	
	*Thamnophis proximus*	Western ribbon snake	
	*Pantherophis slowinskii*	Slowinski’s corn snake	
	*Coluber constrictor*	Eastern racer	
	*Heterodon platirhinos*	Eastern hognose snake	
	*Lampropeltis calligaster*	Prairie kingsnake	
	*Agkistrodon piscivorus*	Western cottonmouth	
